# Overstatements in abstract conclusions claiming effectiveness of interventions in psychiatry: A meta-epidemiological investigation

**DOI:** 10.1371/journal.pone.0184786

**Published:** 2017-09-13

**Authors:** Kiyomi Shinohara, Aya M. Suganuma, Hissei Imai, Nozomi Takeshima, Yu Hayasaka, Toshi A. Furukawa

**Affiliations:** Department of Health Promotion and Human Behavior, Kyoto University Graduate School of Medicine / School of Public Health, Konoe-cho, Sakyo-ku, Kyoto, Japan; University of Illinois-Chicago, UNITED STATES

## Abstract

**Objective:**

Abstracts of scientific reports are sometimes criticized for exaggerating significant results when compared to the corresponding full texts. Such abstracts can mislead the readers. We aimed to conduct a systematic review of overstatements in abstract conclusions in psychiatry trials.

**Methods:**

We searched for randomized controlled trials published in 2014 that explicitly claimed effectiveness of any intervention for mental disorders in their abstract conclusion, using the Cochrane Register of Controlled Trials. Claims of effectiveness in abstract conclusion were categorized into three types: superiority (stating superiority of intervention to control), limited superiority (intervention has limited superiority), and equal efficactiveness (claiming equal effectiveness of intervention with standard treatment control), and full text results into three types: significant (all primary outcomes were statistically significant in favor of the intervention), mixed (primary outcomes included both significant and non-significant results), or all results non-significant. By comparing these classifications, we assessed whether each abstract was overstated. Our primary outcome was the proportion of overstated abstract conclusions.

**Results:**

We identified and included 60 relevant trials. 20 out of 60 studies (33.3%) showed overstatements. Nine reports reported only significant results although none of their primary outcomes were significant. Large sample size (>300) and publication in high impact factor (IF>10) journals were associated with low occurrence of overstatements.

**Conclusions:**

We found that one in three psychiatry studies claiming effectiveness in their abstract conclusion, either superior to control or equal to standard treatment, for any mental disorders were overstated in comparison with the full text results. Readers of the psychiatry literature are advised to scrutinize the full text results regardless of the claims in the abstract.

**Trial registration:**

University hospital Medical Information Network (UMIN) Clinical Trials Registry (UMIN000018668)

## Introduction

Abstracts of scientific articles deliver the most essential findings from the research. In fact, they are the primary source of information for many readers because of their accessibility and conciseness [1, 2]. In particular, conclusion sections of abstracts convey key messages of the research. Consequently, eye-catching abstracts attract many readers, and authors are often under pressure to produce abstracts with interesting, positive findings [3].

However, there is mounting criticism that some abstract conclusions of scientific articles present the results of a randomized controlled trial (RCT) too strongly in favor of the investigation of interest. That is, some abstracts emphasize beneficial effects of intervention beyond the actual findings mentioned in the corresponding full texts. Such distortion can have detrimental impacts, for readers may read abstracts only and take abstract conclusions at face value. Even if readers read entire articles, their interpretation of the articles may be anchored by the exaggerated reporting of abstract conclusions.

Despite the recent efforts to promote transparent reporting, studies have found that many of the abstracts fall short of a standard specifically stated in the new CONSORT guideline [4]. The first review of this problem was provided by Boutron et al [5]. They evaluated each of 72 randomized controlled trials (RCTs) with non-significant primary outcomes through their consensus judgment based on the following definition of ‘spin’: (i) a focus on statistically significant results, (ii) interpreting non-significant results as showing treatment equivalence or comparable effectiveness, and (iii) claiming or emphasizing the beneficial effect of treatment despite non-significant results. According to the study, about 60% of the cases were classified as having spins and spins were most commonly found in abstract conclusions. Subsequently, several studies examined spins in trial reports from various subspecialties [6–8] or in observational studies [9] and even in press prelease by academia [10, 11]. A focus on positive findings was also found in biological studies, leading to more citation of positive results or spun negative studies [12, 13]. Consequently, together with the citation bias, these exaggerations can mislead the press, clinicians and researchers, and poses an obstacle to future research [12–14].

While these studies shed some light on the prevalence of distorted reporting in abstracts, they, however, have limitations. First of all, spin as defined by these investigators may not be free from the subjective and arbitrary viewpoint of investigators and potentially leave room for debate [15]. We need a more objective and systematic approach for a proper assessment of the magnitude of this problem. Second, each of previous studies was limited to specific subspecialties such as rheumatology [7], wound research [16], surgery [8] or to limited topics such as early psychosis trials [6]. As far as we know, there has been no study that evaluated overstatements in psychiatry trial to inform the readers what proportion of abstract conclusions they read may be exaggerated.

This study aimed to evaluate the prevalence and patterns of overstated abstract conclusions in trials claiming effectiveness of interventions, either superior to control or equal to standard treatment for any mental disorders, by systematically and separately evaluating their abstract conclusions and the results of corresponding full text. In addition, we examined the predictors of overstatements.

## Materials and methods

The details of the method can be found in the published protocol [17]. Neither methods or outcomes have been changed critically from our protocol. The only amendment was that we added multivariable logistic regression about factors that had significant association with overstatement. Since we used secondary data from published trial reports, this trial require no ethical approval. This protocol was registered in the University hospital Medical Information Network (UMIN) Clinical Trials Registry (UMIN000018668).

### Study selection

We (KS, AMS) searched Cochrane Central Register of Controlled Trials (CENTRAL) to identify all RCTs claiming effectiveness of intervention for mental disorders published in the English language in 2014 (search data: August 2015) We used the MeSH term ‘mental disorders’ Mesh-term sub-headings ‘drug therapy’ and ‘therapy’, and publication type ‘randomised controlled trial’ (S1 Table).

The selection covered any kinds of interventions, from common pharmacological intervention to non-drug therapy such as aromatherapy and exercise. We included published reports whose abstract conclusions claimed superior or equal effectiveness of intervention to control. ‘Equal effectiveness’ meant that they declared the intervention was equally effective as the standard treatment for the targeted mental illness in their abstract conclusions. We focused on the primary (if stated) or all outcomes (if none was declared primary) in the abstract conclusions. We excluded those reports that explicitly declared the intervention has not superior to the control or effective as standard treatment on their primary outcomes in the abstract conclusion (e.g. ‘the treatment has no significant effect on the primary outcome, depression’), because it is highly unlikely general readers considers those abstract conclusions as claiming effectiveness. We excluded unstructured studies because it would be impossible to determine the conclusion section of them. Trials with more than two arms were also excluded because they would lead to multiple evaluations between different arms. Secondary analysis studies, feasibility studies, and cost effectiveness studies were not included either because their aims do not lie in the evaluation of treatment effectiveness.

### Data extraction

**Fig 1:**
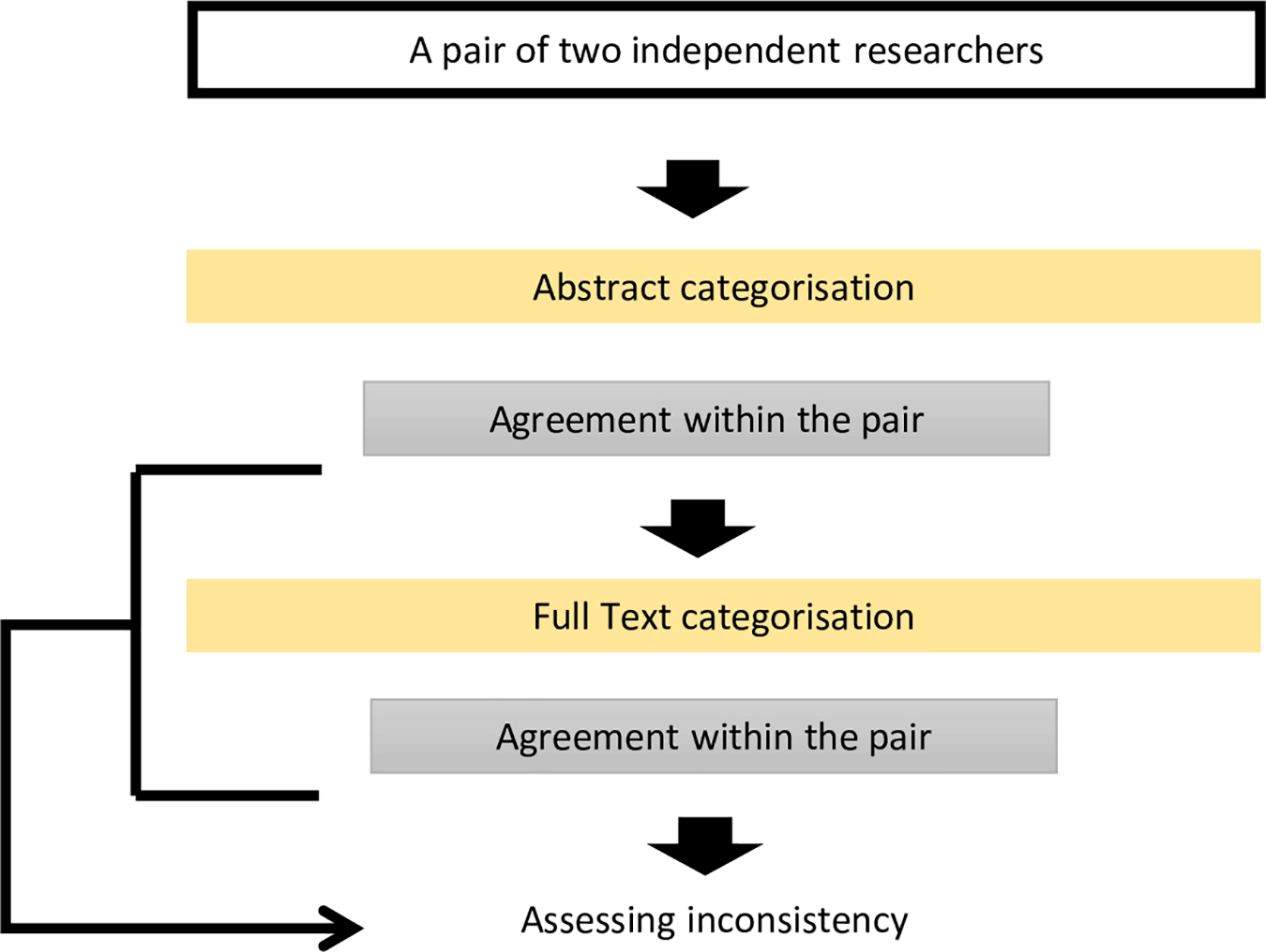
Flow chart of assessment of selected studies.

Two independent researchers identified the eligible studies. Any disagreement was resolved through discussion and in consultation with a third member of the research team. A pair of researchers, who have not screened and therefore have not read the abstract and the paper, independently collected information from each study in the following three steps: categorization of abstracts, classification of primary outcomes, and assessing inconsistency between the two. The agreement within each pair was reported at each step of assessments (Fig 1).

This was to maintain the independence of appraisal from any influence of full text on abstract and vice versa.

We extracted the relevant data from each study using the Excel spreadsheet specially made for this study. The data included: the type of intervention, targeted mental illness, the region where the study was conducted, the number of randomized patients, study design, primary outcomes supposed in abstract conclusion, the results of actual primary and secondary outcomes in the full text. Data extraction, categorization of abstracts, and the evaluation of the primary outcomes were done independently by the two teams consisting of two or three assessors. Any disagreements in the team were resolved by the discussions with a third member of the team (TAF). Citation was recorded in each study PDF document and kept as a reference.

### Classification of abstract conclusions

**Fig 2:**
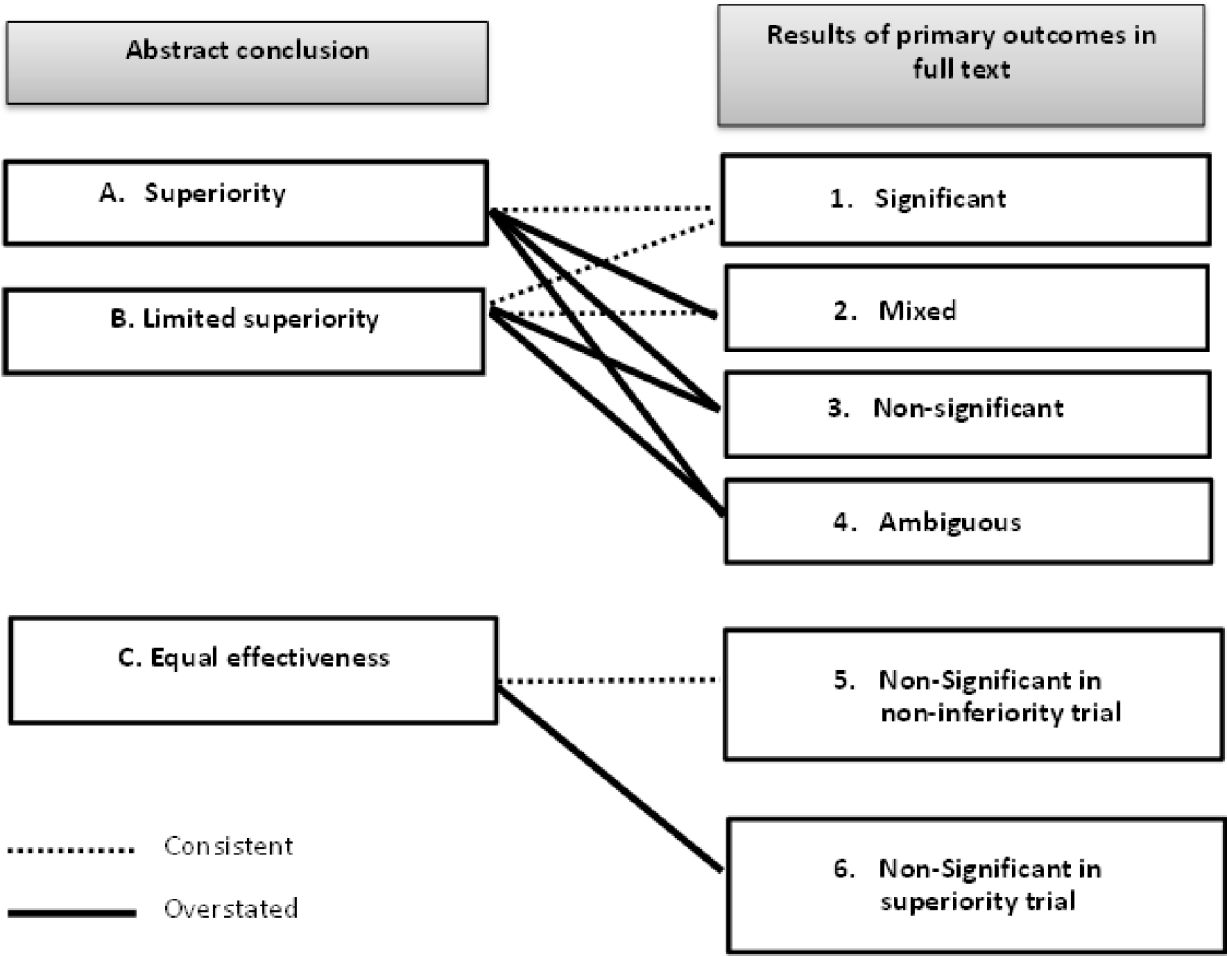
Definitions of overstatement in abstract conclusion.

First, we (KS, YH, NT, HI, AMS) categorized each abstract conclusion into one of the three types according to the level of effectiveness it claimed without reading their full text (Fig 2). When a trial only stated significant effectiveness of intervention in its abstract conclusion, it was classified as ‘superiority’. On the other hand, a trial reporting both significant and non-significant findings with respect to intervention’s effectiveness was considered as ‘limited superiority’ (e.g. ‘treatment significantly improved quality of life than the control, but not depression’, ‘treatment enhanced the rates of recovery, with the effect limited to patients with severe depression’, or ‘treatment improved quality of life and anxiety, and had limited effect on depression’). Trials claiming equal effectiveness of the intervention to the standard treatment (e.g. ‘intervention A was equally effective as standard treatment B’) were categorized as ‘equal effectiveness’. Note that our judgment was based solely on the abstract conclusion, regardless of the primary outcome results discussed in the results section of abstract or full text.

### Classification of results of primary outcome in the full text

Secondly, we (KS, YH, NT, HI, AMS) assessed the level of statistical evidence for their findings in primary outcome(s) in full text, and categorized them into one of the three: significant (all primary outcomes were statistically significant), mixed (primary outcome included statistically significant and non-significant results), and all non-significant. Note that no results of any secondary analysis or subgroup analysis results were taken into consideration when determining the category.

We defined those trials as having ‘ambiguous primary outcome’ if they did not explicitly state any outcome(s) as ‘primary’ or ‘main analysis’, except when they only measured a single outcome. In such cases, the single outcome was considered as primary. When a trial did not specify the primary time point, end of treatment was regarded as primary in trials studying the effectiveness of the acute treatment. We also examined if a trial was designed as superiority or non-inferiority trial.

Furthermore, we defined a trial lacking vital information as ‘sub-quality trial’, such as lacking statistical analysis of the main comparison, and having no assessment at the end of the treatment.

### Assessing inconsistency between abstract and results in full text

Using our approach, overstated abstracts were deduced systematically by comparing the classification of the abstract conclusion and that of the full text for each study as shown in Fig 2. Naturally, a trial with abstract conclusion categorized as ‘superiority’ should have statistically significant results in all of their declared primary outcomes. Similarly, a ‘limited superiority’ abstract conclusion should correspond to mixed results (they should have significant results in at least one primary outcome). ‘Equal effectiveness’ abstract conclusion category must only be supported by the full text describing non-inferiority trials showing effectiveness of treatment at least as much as the control or worse only by a pre-specified amount. Note that by definition, non-inferiority trials are designed and conducted using a specific methodology under a specific design, such as the sample size calculation and equivalence margin pre-specification. The full text results should show that the upper limit of 95% confidence intervals (CIs) for the difference between intervention and control lies below that equivalence margin [18]. For a study that did not fall into any of the above patterns, it is regarded as having an overstated abstract conclusion.

### Outcomes of the current study

#### Primary outcome

Our primary outcome was the proportion of overstated abstract conclusions out of all the studies that had claimed effectiveness in their abstract conclusions. Fig 2 shows correspondences between abstract conclusions and results of primary outcomes in full text. Our primary outcome is then the sum of studies in the categories A-2, A-3, A-4, B-3, B-4 and C-6, divided by all the included studies.

#### Secondary outcome

We next examined which of the abstracts, stating ‘superiority’ (category A in Fig 2) or stating ‘limited superiority’ (category B), are more likely to be overstating the primary outcomes in their full texts. This was examined by calculating the risk ratio (RR) of the proportion of explicit overstatement among the ‘superiority’ category (A-2 + A-3 over A) over that among the ‘limited superiority’ category (B-3 over B).

#### Sub-group analyses

We investigated factors that can potentially be associated with inconsistencies, such as impact factors (IF) of the journal in which the trials are published, source(s) of funding, and the sample size. For our purposes, top ten impact factor journals as ranked in *Journal Citation Report (2014)* in general medicine, psychiatry, and psychology were regarded as high IF journals. The differences between the groups were checked by Fisher’s exact test for dichotomous data, and Mann-Whitney *U*- test for continuous data. We used SPSS statistics 22 (SPSS Japan Inc., Tokyo, Japan) and considered p<0.05 (two-sided) as significant. If any factor had significant association with overstatement, we calculated the odds ratio by using logistic regression analysis. We excluded sub-quality trials when conducting this sub-group analysis because we could not tell whether they overstated the result or not.

## Results

**Fig 3:**
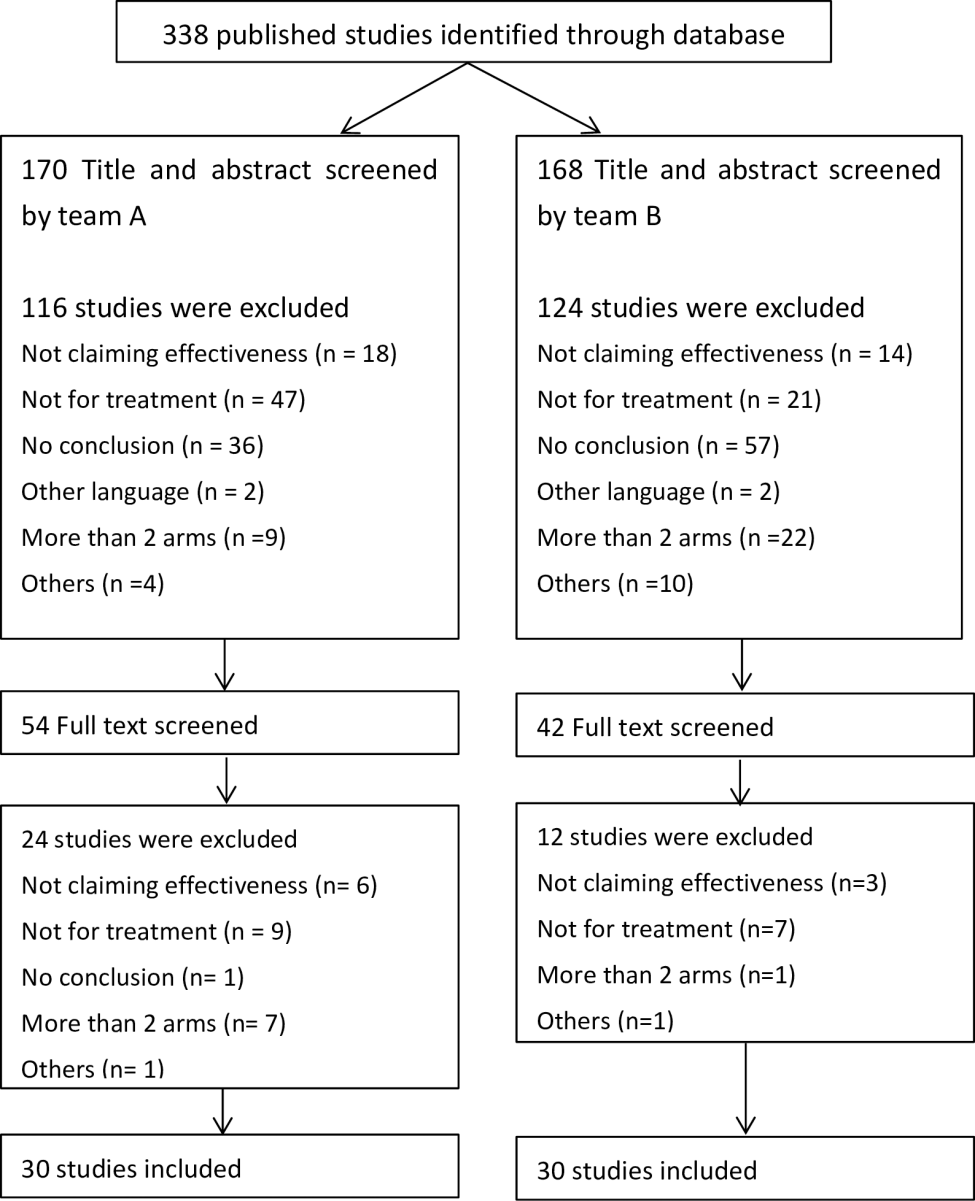
Flow chart of study selection.

Through our electronic search in the Cochrane CENTRAL, we identified 338 published studies, and 60 studies were included in total after screening by two pairs of investigators (See S2 for the list of all included trials). 93 studies were excluded due to the lack of a conclusion section in their abstracts (Fig 3). Table 1 shows the characteristics of the included studies. The sample size ranged widely (mean: 199, median 131, range 21 to 1310).

**Table 1:** Characteristics of trials (n = 60).

Characteristics		Total number of Trials titirald	%
Journal	High IF journals	26	43.3
	Other IF journals	34	56.7
Region	North America	37	61.7
	Europe	14	23.3
	Cross-continental	2	3.3
	Africa	2	3.3
	Asia	2	3.3
	Oceania	1	1.7
Interventions	Non-drug therapy	36	60.0
	Drug therapy	16	26.7
	Combination	8	13.3
Sponsorship by for-profit entity	No	48	80.0
	Yes	9	15.0
	Unclear	3	5.0
Design	Superiority	58	96.7
	Non-inferiority	2	3.3
Included disorders	Problems related to substance use disorders	19	31.7
	Mood disorders	11	18.3
	Cognitive disorders	4	6.7
	Schizophrenia	2	3.3
	Others	24	40.0

### Categorization of abstracts and full text

In the abstract conclusion, 44 (73.3%) studies claimed superior effectiveness of interventions compared to control, 13 (21.7%) studies stated limited effectiveness, and three (5.0%) studies claimed equal effectiveness. On the other hand, in the full text, only 30 (50.0%) studies showed statistically significant difference in all primary outcomes, 13 (21.7%) studies had mixed results (significant and non-significant), and nine (15.0%) studies, all primary outcomes were non-significant. Three (5%) studies did not declare their primary outcomes in the full text (Table 2). Our inter-rater reliability was a kappa of 0.62 (95% CI: 0.40 to 0.85) for classification of abstract conclusion, and a kappa of 0.83 (95% CI: 0.71 to 0.94) for classification of full text results.

**Table 2:** Summary of results: Overstatements (shaded cells) in abstract conclusion (N = 60(%)).

Full text results (rows) Abstract conclusion (columns)	Statistically Significant	Mixed	Non-significant	Ambiguous	Sub-quality	Non-inferiority	Superiority	Total
Superiority	29	7	4	3	1	-	-	44
	(48.0)	(11.7)	(6.7)	(5.0)	(1.7)			
Limited superiority	1	6	5	0	1	-	-	13
	(1.7)	(10.0)	(8.3)	(0.0)	(1.7)			
Equal effectiveness	-	-	-	-	-	2	1	3
						(3.3)	(1.7)	
								60

Comparing these abstract conclusions and the results of the primary outcomes, 20 (33.3%) studies overstated their results in the abstract, while 38 (63.3%) did not. Two studies were classified as ‘sub-quality’ because they only reported the results of subgroup analysis. Ten studies claimed superiority or limited superiority of intervention even though all of their primary outcomes were statistically non-significant. (S2 Table provides details of overstatements). There was not a significant difference in our secondary outcomes, the proportion of explicit overstatement, among ‘superiority’ studies (11 out of 44) and ‘limited superiority’ studies (5 out of 13) (RR; 0.82, 95% CI, 0.35–1.93). The strategy of overstatement varied. For example, some trials with no significant primary outcomes failed to refer to non-significant results and claimed effectiveness by focusing on significant results from subgroup analyses or secondary outcomes [19, 20]. Some studies acknowledged that the outcomes were non-significant [21–23] or suggested that ‘replication is needed’ [24]; nevertheless, they still emphasized significant secondary outcomes or used phrases such as ‘… is promising treatment’ to overstate the results.

### Study characteristics associated with overstatement

**Fig 4:**
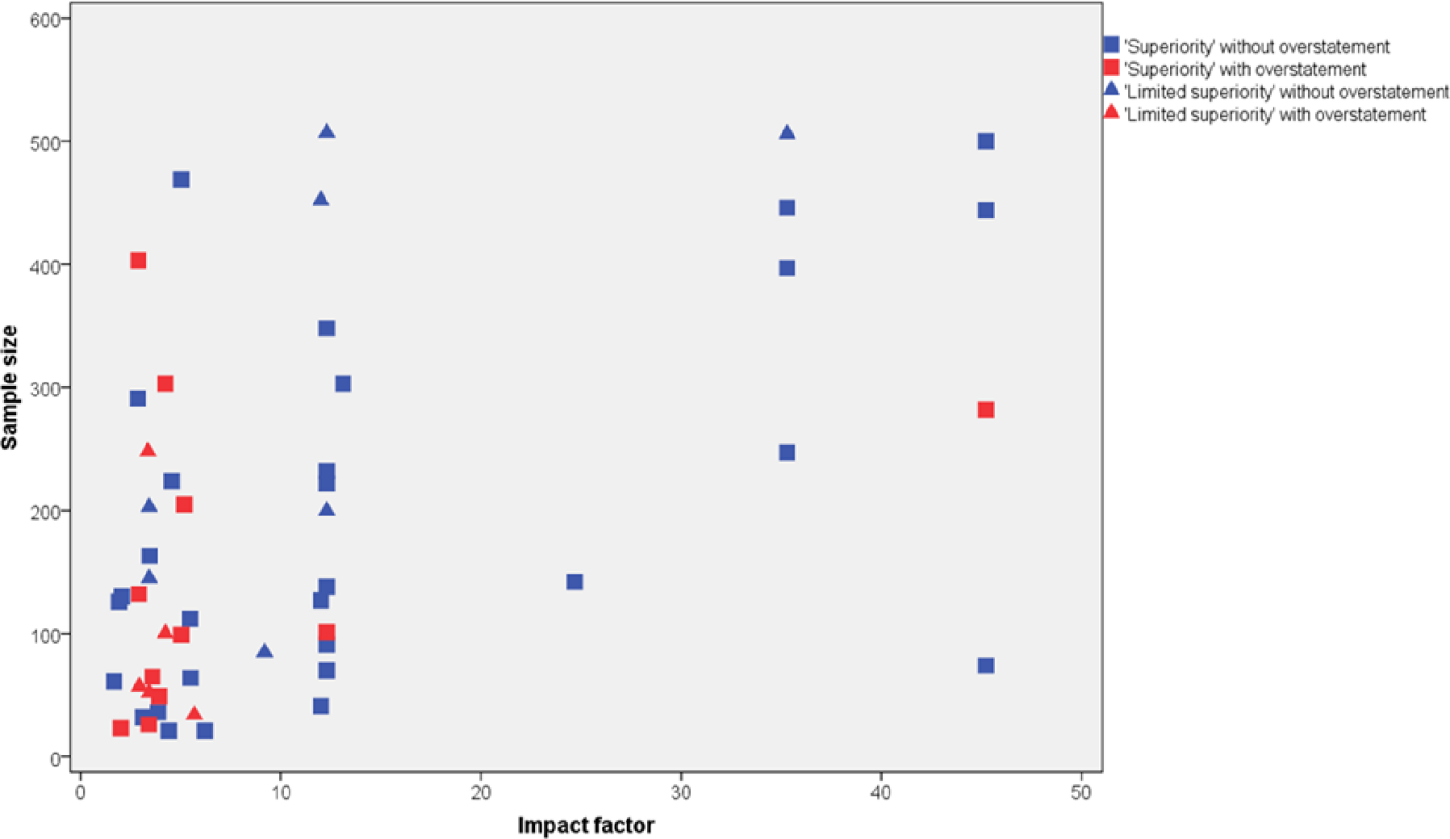
Relation between sample size, impact factor of journal, and overstatements in ‘superiority’ and ‘limited superiority’ trials.

Sample size and impact factor of journals were significantly associated with the prevalence of overstatement (Fig 4). Studies with smaller sample size overstated more than those with larger sample size (by Mann-Whitney U- test, p = 0.049). Overstatements were less common in studies published in high impact factor journals than otherwise (the risk ratio was 0.15, 95% CI: 0.04 to 0.57). There were no significant association between sponsorship and overstated abstracts. In the multivariable logistic regression, entering associated factors together (sample size and high IF), the odds ratio of larger sample size for overstatement was 0.86 per 100 persons (95% CI: 0.53 to 1.39), and that of high IF journals was 0.09 (95% CI: 0.02 to 0.47) vs low IF journals.

## Discussion

The systematic comparison between the claims in the abstract conclusions and the findings of the primary outcomes in the full text revealed that 32% (14/44) of the claims for clear effectiveness of interventions for mental disorders were overstated in the abstract. Including abstracts claiming limited effectiveness or equal effectiveness, 33% (20/60) were judged to be cases of overstatement. Five studies were judged to represent sub-quality or ambiguous reportings.

By and large these figures are comparable to those reported for other subspecialties [6–8]. In other words, psychiatry fared neither better nor worse than other medical fields such as rheumatology, surgery or wound medicine. In line with a previous study which found that high impact factor and small sample size were associated with better conduct and reporting of psychiatry trials [25], our results showed that small sample size and low impact factors of journals may be suggestive of the existence of overstatements. Journals with IF less than 10 and/or trials with less than 300 participants were particularly likely to overstate the findings (Fig 4).

We faced some challenges while conducting this study that may speak for further weaknesses of the literature beyond the overstatements that we studied. For instance, we found non-structured abstracts were common in psychiatry trial reports (93/338, 28%). This is because some journals still have not introduced structured abstracts as recommended by the CONSORT statement that requires structured abstracts when reporting RCTs [26]. In addition, some of the studies reported the conclusion in ambiguous language, such as a treatment ‘may be effective [19, 27]’, or has ‘the potential [28]’. Such expressions may leave impressions that are not only different from the study’s actual findings, but also highly variable among the readers.

### Strengths and limitations

The strong points of this study may be as follows. First, this study is the first systematic analysis of overstatements in the psychiatry trial literature. We have revealed that distorted abstract reporting is a fairly common practice even in psychiatry. Second, our approach is more structured than in the previous studies. In our framework, overstatements are identified through systematic evaluations of abstracts and full texts separately, and not by any overall, subjective decision call by investigators who are actively gleaning spins. Third, we adopted the viewpoint of general consumers as they would first read abstracts, and were able to provide some practical tips in navigating though the psychiatry literature.

This study also has several limitations. Although we minimized investigators presumption and prejudice in evaluation, there still was some room left for judgment for the categorization of abstract conclusions and full text results. Still, we were able to achieve high inter-rater reliability in these judgments when we followed our pre-specified protocol nonetheless. Second, this approach has not been tested outside of mental disorders, and may require some modification when it is applied to other fields. Lastly, the present study did not address the question of how much impact the overstatements actually have on the readers’ interpretation of study results. We are currently investigating this issue in a randomized controlled trial.

### Implication for researchers

Despite the new guidelines [4, 29], distorted abstract reporting is regrettably a common practice, and researchers should take into consideration the impact of abstract reporting, and communicate their findings of the primary outcome in a plain direct style.

### Implication for journal editors and peer reviewers

Journal editors should require that authors abide by the CONSORT guideline by providing specific writing instructions. Furthermore, journal editors and reviewers should ensure that abstracts of a paper are not overstated.

### Implication for consumers of the medical literature

One third of published psychiatry articles claiming effectiveness of interventions, either superior to the control or equal to the standard treatment had overstatement in abstract conclusions. While sample size and impact factors may suggest some indication, there still is good probability of overstatements even in plausibly good articles. Because abstract conclusions in themselves reveal very little about the possible existence of overstatements, consumers of scientific literature should read articles while always being mindful of specified primary and secondary outcomes in the full text.

## Supporting information

S1 Table Search terms to identify related studies. (DOCX)

S2 Table The patterns of overstatement and sub-quality trials. (DOCX)

S1 File The list of all included trials. (DOCX)
