# Supplementary material for: Overstatements in abstract conclusions claiming effectiveness of interventions in psychiatry: A meta-epidemiological investigation
Source: PLoS One. 2017 Sep 13;12(9):e0184786. doi: 10.1371/journal.pone.0184786 (PMC5597227; doi:10.1371/journal.pone.0184786)
Supplement: S1 Table — (DOCX) [file pone.0184786.s001.docx]

S1 Table. Search terms to identify related studies.

| **Search Terms** | |
| --- | --- |
| #1 | MeSH descriptor: [Mental Disorders] explode all trees and with qualifier(s): [Therapy - TH] |
| #2 | MeSH descriptor: [Mental Disorders] explode all trees and with qualifier(s): [Drug therapy - DT] |
| #3 | "randomised controlled trial":pt Publication Year from 2014 to 2014 (Word variations have been searched) |
| #4 | (#1 or #2) and #3 |
