# Supplementary material for: Overstatements in abstract conclusions claiming effectiveness of interventions in psychiatry: A meta-epidemiological investigation
Source: PLoS One. 2017 Sep 13;12(9):e0184786. doi: 10.1371/journal.pone.0184786 (PMC5597227; doi:10.1371/journal.pone.0184786)
Supplement: S2 Table — (DOCX) [file pone.0184786.s002.docx]

S2 Table. The patterns of overstatement and subquality trials (numbers above the table are reference ID)

27

| Title | Counselor-assisted problem solving (CAPS) improves behavioral outcomes in older adolescents with complicated mild to severe TBI |
| --- | --- |
| Conclusion | Online problem-solving therapy may be effective in reducing behavior problems in older adolescent survivors of moderate-severe TBI. |
| Classification of Abstract | Superior |
| Classification of results | NS |
| Primary outcomes and results | Child Behavior Checklist (CBCL) administered before and after completion of treatment: NS |

29

| Title | Randomized placebo-controlled D-cycloserine with cognitive behavior therapy for pediatric posttraumatic stress |
| --- | --- |
| Conclusion | This initial study of CBT and DCS to treat pediatric PTSD provided suggestive and preliminary evidence for more rapid symptom recovery and beneficial effects on attention, but did not show an overall greater effect for reducing PTSD symptoms. It appears that augmentation with DCS represents unique challenges in PTSD. Because PTSD involves complex, life- threatening trauma memories, as opposed to the imagined dreadful outcomes of other anxiety disorders, the use of DCS may require greater attention to how its use is coupled with exposure-based techniques. DCS may have inadvertently enhanced reconsolidation of trauma memories rather than more positive and adaptive memories. In addition, the results suggest that future research could focus on the longer-term benefits of DCS on attention and ways to capitalize on attention-enhancing therapies. |
| Classification of Abstract | limited |
| Classification of results | NS |
| Primary outcomes and results | CPSS:NS |

40

| Title | Effects of post-session administration of methylene blue on fear extinction and contextual memory in adults with claustrophobia |
| --- | --- |
| Conclusion | Methylene blue enhances memory and the retention of fear extinction when administered after a successful exposuresession but may have a deleterious effect on extinction when administered after an unsuccessful exposure session. |
| Classification of Abstract | Limited |
| Classification of results: | Subquality |
| Primary outcomes and results | Peak fear 1 month later |

69

| Title | Collaborative care outcomes for pediatric behavioral health problems: a cluster randomized trial |
| --- | --- |
| Conclusion | Implementing a collaborative care intervention for behavior problems in community pediatric practices is feasible and broadly effective, supporting the utility of integrated behavioral health care services. |
| Classification of Abstract | Superior |
| Classification of results | Ambiguous PO |
| Primary outcomes and results | Ambiguous PO |

78

| Titles | Attentional retraining administered in the field reduces smokers' attentional bias and craving |
| --- | --- |
| Conclusion | AR can be administered on a mobile device in the natural environment, and AR can reduce attentional bias and craving. |
| Classification of Abstract | Superior |
| Classification of results: | Mixed |
| Primary outcomes and results | Attentional bias : mixed, cued craving: mixed |

145

| Title | A multifaith spiritually based intervention versus supportive therapy for generalized anxiety disorder: a pilot randomized controlled trial |
| --- | --- |
| Conclusion | This small pilot trial demonstrates that a nondenomina- tional SBI has greater efficacy than a rigorous control in improving symptoms of GAD and enhancing spiritual well-being. These results are encouraging and further research on the efficacy of the SBI and its underlying mechanisms is warranted. |
| Classification of Abstract | Superior |
| Classification of results | Mixed |
| Primary outcomes and results | HAM-A ; SS, PSWQ:SS, BAI;NS |

189

| Title | Parent-implemented social intervention for toddlers with autism: an RCT |
| --- | --- |
| Conclusion | These findings support the efficacy of individual-ESI compared with group-ESI on child outcomes, suggesting the importance of individualized parent coaching in natural environments. The efficacy of a parent-implemented intervention using little professional time has potential for community viability, which is particularly important in light of the lack of main effects on child outcomes of most other parent-implemented interventions. |
| Classification of Abstract | Superior |
| Classification of results: | Ambiguous PO |
| Primary outcomes and results | Ambiguous PO |

235

| Title | The cognitive behavioural prevention of suicide in psychosis: a clinical trial |
| --- | --- |
| Conclusion | CBSPp is a feasible intervention which has the potential to reduce proxy measures of suicide in psychotic patients. |
| Classification of Abstract | Superior |
| Classification of results: | Mixed |
| Primary outcomes and results | The Beck Scale for Suicidal ideation(BSS): NS, The Adult Suicidal Ideation Questionnaire: SS, The Suicide Probability Scale(SPS):SS |

240

| Title | Stepped care in the treatment of trichotillomania |
| --- | --- |
| Conclusion | Considering the entire stepped care program, participants significantly reduced symptoms, alopecia, and impairment, and increased quality of life. For quality of life and symptom severity, there was some relapse by 3-month follow-up. CONCLUSIONS: Stepped care is acceptable, and HRT was associated with improvement. Further work is needed to determine which patients with TTM can benefit from self-help and how to reduce relapse. |
| Classification of Abstract | Superior |
| Classification of results | Ambiguous PO |
| Primary outcomes and results | Ambiguous PO |

270

| Title | Comparative short term efficacy and tolerability of methylphenidate and atomoxetine in attention deficit hyperactivity disorder |
| --- | --- |
| Conclusion | Methylphenidate and atomoxetine are efficacious in Indian children with ADHD at lesser doses than previously used. Their efficacy and tolerability are comparable. |
| Classification of Abstract | Equal |
| Classification of results | NS in superiority design |
| Primary outcomes and results | Improvement in symptoms as assessed by VADPRS: NS |

307

| Title | A pilot randomised trial to assess the methods and procedures for evaluating the clinical effectiveness and cost-effectiveness of Exercise Assisted Reduction then Stop (EARS) among disadvantaged smokers |
| --- | --- |
| Conclusion | The study provided valuable information on the resources needed to improve study recruitment and retention. Offering support for smoking reduction and PA appears to have value in promoting reduction and cessation in disadvantaged smokers not currently motivated to quit. A large RCT is needed to assess the clinical effectiveness and cost-effectiveness of the intervention in this population. |
| Classification of Abstract | Superior |
| Classification of results | NS |
| Primary outcomes and results | Confirmed quit at week 4: NS |

19

| Title | A randomized pilot clinical trial to evaluate the efficacy of Community Reinforcement and Family Training for Treatment Retention (CRAFT-T) for improving outcomes for patients completing opioid detoxification |
| --- | --- |
| Conclusion | Conclusion: CRAFT-T is a promising treatment for opioid use disorder but replication is needed to confirm these results. |
| Classification of Abstract | Limited |
| Classification of results: | NS |
| Primary outcomes and results | Time to drop-out: NS |

53

| Title | Results of the citalopram to enhance cognition in Huntington disease trial |
| --- | --- |
| Conclusion | Conclusions: There was no evidence that short-term treatment with citalopram improved executive functions in HD. Despite excluding patients with active depression, participants on citalopram showed improved mood, raising the possibility of efficacy for subsyndromal depression in HD. |
| Classification of Abstract | limited |
| Classification of results | NS |
| Primary outcomes and results | changes in executive composite score: NS |

59

| Title | A pilot randomized controlled trial of Dialectical Behavior Therapy with and without the Dialectical Behavior Therapy Prolonged Exposure protocol for suicidal and self-injuring women with borderline personality disorder and PTSD |
| --- | --- |
| Conclusion | There was no evidence that short-term treatment with citalopram improved executive functions in HD. Despite excluding patients with active depression, participants on citalopram showed improved mood, raising the possibility of efficacy for subsyndromal depression in HD. |
| Classification of Abstract | superior |
| Classification of results | NS |
| Primary outcomes and results | PTSD: NS |

127

| Title | Improving care for depression in obstetrics and gynecology: a randomized controlled trial |
| --- | --- |
| Conclusion | Collaborative depression care adapted to women's health settings improved depressive and functional outcomes and quality of depression care." |
| Classification of Abstract | Superior |
| Classification of results | mixed |
| Primary outcomes and results | reduction in depression symptom at12 months: SS  functional status at 12 months: NS |

134

| Title | Efficacy of exercise for menopausal symptoms: a randomized controlled trial |
| --- | --- |
| Conclusion | These findings provide strong evidence that 12-weeks of moderate-intensity aerobic exercise does not alleviate VMS but may result in small improvements in sleep quality, insomnia and depression in midlife, sedentary women. |
| Classification of Abstract | Limited |
| Classification of results: | NS |
| Primary outcomes and results | Vasomotor symptom decrease: NS, vasomother frequency: NS |

135

| Title | A multidimensional home-based care coordination intervention for elders with memory disorders: the maximizing independence at home (MIND) pilot randomized trial |
| --- | --- |
| Conclusion | A home-based dementia care coordination intervention delivered by non-clinical community workers trained and overseen by geriatric clinicians led to delays in transition from home, reduced unmet needs, and improved self-reported QOL. |
| Classification of Abstract | Superior |
| Classification of results | Mixed |
| Primary outcomes and results | likelihood of leaving home permanently; SS, total percent of unmet care needs at 18 months (NS) |

210

| Title | Effectiveness of nurse-practitioner-delivered brief motivational intervention for young adult alcohol and drug use in primary care in South Africa: a randomized clinical trial |
| --- | --- |
| Conclusion | Brief Motivational Intervention may be effective at reducing at-risk alcohol use in the short term among low-income young adult primary care patients; additional research is needed to examine long-term outcomes. |
| Classification of Abstract | Superior |
| Classification of results | mixed |
| Primary outcomes and results | ASSIST scores: mixed |

213

| Title | Computer-assisted delivery of cognitive-behavioral therapy: efficacy and durability of CBT4CBT among cocaine-dependent individuals maintained on methadone |
| --- | --- |
| Conclusion | This trial replicates earlier findings indicating that CBT4CBT is an effective adjunct to addiction treatment with durable effects. CBT4CBT is an easily disseminable strategy for broadening the availability of CBT, even in challenging populations such as cocaine-dependent individuals enrolled in methadone maintenance programs. |
| Classification of Abstract | superior |
| Classification of results | Mixed |
| Primary outcomes and results | Percent days of abstinence, self-report: NS, 3 or more weeks of continuous abstinence: SS |

253

| Title | Galantamine versus risperidone treatment of neuropsychiatric symptoms in patients with probable dementia: an open randomized trial |
| --- | --- |
| Conclusion | Conclusions: These results support that galantaine, with its benign safety profile, can be used as first-line treatment of NPSD symptoms, unlsess symptoms of irritation and agitation are prominent, where risperidone is more efficient. |
| Classification of Abstract | limited |
| Classification of results: | NS |
| Primary outcomes and results | NSI Scores: NS |

282

| Title | Effectiveness of a community-based intervention for people with schizophrenia and their caregivers in India (COPSI): a randomised controlled trial |
| --- | --- |
| Conclusion | The collaborative community-based care plus facility-based care intervention is modestly more effective than facility-based care, especially for reducing disability and symptoms of psychosis. Our results show that the study intervention is best implemented as an initial service in settings where services are scarce, for example in rural areas. |
| Classification of Abstract | Superior |
| Classification of results | NS |
| Primary outcomes and results | Change in symptoms and disabilities in 12 months: NS |
