# Supplementary material for: Overstatements in abstract conclusions claiming effectiveness of interventions in psychiatry: A meta-epidemiological investigation
Source: PLoS One. 2017 Sep 13;12(9):e0184786. doi: 10.1371/journal.pone.0184786 (PMC5597227; doi:10.1371/journal.pone.0184786)
Supplement: S1 File — (DOCX) [file pone.0184786.s003.docx]

S 1 File. The list of all include trials ([1-60](#_ENREF_1)).

1. Rosenblum A, Matusow H, Fong C, et al. Efficacy of dual focus mutual aid for persons with mental illness and substance misuse. Drug and alcohol dependence. 2014;135:78-87

2. McDonell M, McPherson S, Vilardaga R, et al. Preliminary findings: Contingency management targeting psycho-stimulant use results in secondary decreases in smoking for severely mentally ill adults. The American journal on addictions 2014;23:407-10

3. Brigham GS, Slesnick N, Winhusen TM, et al. A randomized pilot clinical trial to evaluate the efficacy of Community Reinforcement and Family Training for Treatment Retention (CRAFT-T) for improving outcomes for patients completing opioid detoxification. Drug and alcohol dependence. 2014;138:240-3

4. Prochaska JJ, Hall SE, Delucchi K, et al. Efficacy of initiating tobacco dependence treatment in inpatient psychiatry: a randomized controlled trial. American journal of public health. 2014;104:1557-65

5. Wade SL, Stancin T, Kirkwood M, et al. Counselor-assisted problem solving (CAPS) improves behavioral outcomes in older adolescents with complicated mild to severe TBI. The Journal of head trauma rehabilitation. 2014;29:198-207

6. Scheeringa MS, Weems CF. Randomized placebo-controlled D-cycloserine with cognitive behavior therapy for pediatric posttraumatic stress. Journal of child and adolescent psychopharmacology. 2014;24:69-77

7. Gois C, Dias VV, Carmo I, et al. Treatment response in type 2 diabetes patients with major depression. Clinical psychology & psychotherapy. 2014;21:39-48

8. Telch MJ, Bruchey AK, Rosenfield D, et al. Effects of post-session administration of methylene blue on fear extinction and contextual memory in adults with claustrophobia. The American journal of psychiatry. 2014;171:1091-8

9. Kennard BD, Emslie GJ, Mayes TL, et al. Sequential treatment with fluoxetine and relapse--prevention CBT to improve outcomes in pediatric depression. The American journal of psychiatry. 2014;171:1083-90

10. Hollon SD, DeRubeis RJ, Fawcett J, et al. Effect of cognitive therapy with antidepressant medications vs antidepressants alone on the rate of recovery in major depressive disorder: a randomized clinical trial. JAMA psychiatry. 2014;71:1157-64

11. Beglinger LJ, Adams WH, Langbehn D, et al. Results of the citalopram to enhance cognition in Huntington disease trial. Movement disorders 2014;29:401-5

12. Harned MS, Korslund KE, Linehan MM. A pilot randomized controlled trial of Dialectical Behavior Therapy with and without the Dialectical Behavior Therapy Prolonged Exposure protocol for suicidal and self-injuring women with borderline personality disorder and PTSD. Behaviour research and therapy. 2014;55:7-17

13. Kolko DJ, Campo J, Kilbourne AM, et al. Collaborative care outcomes for pediatric behavioral health problems: a cluster randomized trial. Pediatrics. 2014;133:e981-92

14. Kerst WF, Waters AJ. Attentional retraining administered in the field reduces smokers' attentional bias and craving. Health psychology 2014;33:1232-40

15. Evins AE, Cather C, Pratt SA, et al. Maintenance treatment with varenicline for smoking cessation in patients with schizophrenia and bipolar disorder: a randomized clinical trial. JAMA. 2014;311:145-54

16. Melville JL, Reed SD, Russo J, et al. Improving care for depression in obstetrics and gynecology: a randomized controlled trial. Obstetrics and gynecology. 2014;123:1237-46

17. Sternfeld B, Guthrie KA, Ensrud KE, et al. Efficacy of exercise for menopausal symptoms: a randomized controlled trial. Menopause. 2014;21:330-8

18. Samus QM, Johnston D, Black BS, et al. A multidimensional home-based care coordination intervention for elders with memory disorders: the maximizing independence at home (MIND) pilot randomized trial. The American journal of geriatric psychiatry. 2014;22:398-414

19. Tyrer P, Cooper S, Salkovskis P, et al. Clinical and cost-effectiveness of cognitive behaviour therapy for health anxiety in medical patients: a multicentre randomised controlled trial. Lancet. 2014;383:219-25

20. Koszycki D, Bilodeau C, Raab-Mayo K, et al. A multifaith spiritually based intervention versus supportive therapy for generalized anxiety disorder: a pilot randomized controlled trial. Journal of clinical psychology. 2014;70:489-509

21. Kolk BA, Stone L, West J, et al. Yoga as an adjunctive treatment for posttraumatic stress disorder: a randomized controlled trial. The Journal of clinical psychiatry. 2014;75:e559-65

22. Walker J, Hansen CH, Martin P, et al. Integrated collaborative care for major depression comorbid with a poor prognosis cancer (SMaRT Oncology-3): a multicentre randomised controlled trial in patients with lung cancer. The Lancet Oncology. 2014;15:1168-76

23. Schwenk M, Zieschang T, Englert S, et al. Improvements in gait characteristics after intensive resistance and functional training in people with dementia: a randomised controlled trial. BMC geriatrics. 2014;14:73

24. Sharpe M, Walker J, Holm Hansen C, et al. Integrated collaborative care for comorbid major depression in patients with cancer (SMaRT Oncology-2): a multicentre randomised controlled effectiveness trial. Lancet 2014;384:1099-108

25. Monticone M, Ferrante S, Teli M, et al. Management of catastrophising and kinesiophobia improves rehabilitation after fusion for lumbar spondylolisthesis and stenosis. A randomised controlled trial. European spine journal 2014;23:87-95

26. Wetherby AM, Guthrie W, Woods J, et al. Parent-implemented social intervention for toddlers with autism: an RCT. Pediatrics. 2014;134:1084-93

27. Poulsen S, Lunn S, Daniel SI, et al. A randomized controlled trial of psychoanalytic psychotherapy or cognitive-behavioral therapy for bulimia nervosa. The American journal of psychiatry. 2014;171:109-16

28. Oslin DW, Lynch KG, Maisto SA, et al. A randomized clinical trial of alcohol care management delivered in Department of Veterans Affairs primary care clinics versus specialty addiction treatment. Journal of general internal medicine. 2014;29:162-8

29. Mertens JR, Ward CL, Bresick GF, et al. Effectiveness of nurse-practitioner-delivered brief motivational intervention for young adult alcohol and drug use in primary care in South Africa: a randomized clinical trial. Alcohol and alcoholism 2014;49:430-8

30. Carroll KM, Kiluk BD, Nich C, et al. Computer-assisted delivery of cognitive-behavioral therapy: efficacy and durability of CBT4CBT among cocaine-dependent individuals maintained on methadone. The American journal of psychiatry. 2014;171:436-44

31. Choi SH, Waltje AH, Ronis DL, et al. Web-enhanced tobacco tactics with telephone support versus 1-800-QUIT-NOW telephone line intervention for operating engineers: randomized controlled trial. Journal of medical Internet research. 2014;16:e255

32. Wong ML, Dong C, Flores DL, et al. Clinical outcomes and genome-wide association for a brain methylation site in an antidepressant pharmacogenetics study in Mexican Americans. The American journal of psychiatry. 2014;171:1297-309

33. Fazeli PK, Wang IS, Miller KK, et al. Teriparatide increases bone formation and bone mineral density in adult women with anorexia nervosa. The Journal of clinical endocrinology and metabolism. 2014;99:1322-9

34. Tarrier N, Kelly J, Maqsood S, et al. The cognitive behavioural prevention of suicide in psychosis: a clinical trial. Schizophrenia research. 2014;156:204-10

35. Steinglass JE, Albano AM, Simpson HB, et al. Confronting fear using exposure and response prevention for anorexia nervosa: A randomized controlled pilot study. The International journal of eating disorders. 2014;47:174-80

36. Rogers K, Banis M, Falkenstein MJ, et al. Stepped care in the treatment of trichotillomania. Journal of consulting and clinical psychology. 2014;82:361-7

37. Kramer U, Kolly S, Berthoud L, et al. Effects of motive-oriented therapeutic relationship in a ten-session general psychiatric treatment of borderline personality disorder: a randomized controlled trial. Psychotherapy and psychosomatics. 2014;83:176-86

38. Rigotti NA, Regan S, Levy DE, et al. Sustained care intervention and postdischarge smoking cessation among hospitalized adults: a randomized clinical trial. JAMA. 2014;312:719-28

39. Campbell AN, Nunes EV, Matthews AG, et al. Internet-delivered treatment for substance abuse: a multisite randomized controlled trial. The American journal of psychiatry. 2014;171:683-90

40. Kranzler HR, Covault J, Feinn R, et al. Topiramate treatment for heavy drinkers: moderation by a GRIK1 polymorphism. The American journal of psychiatry. 2014;171:445-52

41. Freund-Levi Y, Jedenius E, Tysen-Bäckström AC, et al. Galantamine versus risperidone treatment of neuropsychiatric symptoms in patients with probable dementia: an open randomized trial. The American journal of geriatric psychiatry. 2014;22:341-8

42. Walker N, Howe C, Glover M, et al. Cytisine versus nicotine for smoking cessation. The New England journal of medicine. 2014;371:2353-62

43. Alaka KJ, Noble W, Montejo A, et al. Efficacy and safety of duloxetine in the treatment of older adult patients with generalized anxiety disorder: a randomized, double-blind, placebo-controlled trial. International journal of geriatric psychiatry. 2014;29:978-86

44. Liebowitz MR, Salman E, Nicolini H, et al. Effect of an acute intranasal aerosol dose of PH94B on social and performance anxiety in women with social anxiety disorder. The American journal of psychiatry. 2014;171:675-82

45. Garg J, Arun P, Chavan BS. Comparative short term efficacy and tolerability of methylphenidate and atomoxetine in attention deficit hyperactivity disorder. Indian pediatrics. 2014;51:550-4

46. Freeman J, Sapyta J, Garcia A, et al. Family-based treatment of early childhood obsessive-compulsive disorder: the Pediatric Obsessive-Compulsive Disorder Treatment Study for Young Children (POTS Jr)--a randomized clinical trial. JAMA psychiatry. 2014;71:689-98

47. Chatterjee S, Naik S, John S, et al. Effectiveness of a community-based intervention for people with schizophrenia and their caregivers in India (COPSI): a randomised controlled trial. Lancet 2014;383:1385-94

48. Iacoviello BM, Wu G, Alvarez E, et al. Cognitive-emotional training as an intervention for major depressive disorder. Depression and anxiety. 2014;31:699-706

49. Kasari C, Lawton K, Shih W, et al. Caregiver-mediated intervention for low-resourced preschoolers with autism: an RCT. Pediatrics. 2014;134:e72-9

50. Morrison AP, Turkington D, Pyle M, et al. Cognitive therapy for people with schizophrenia spectrum disorders not taking antipsychotic drugs: a single-blind randomised controlled trial. Lancet 2014;383:1395-403

51. Hantsoo L, Ward-O'Brien D, Czarkowski KA, et al. A randomized, placebo-controlled, double-blind trial of sertraline for postpartum depression. Psychopharmacology. 2014;231:939-48

52. Tannenbaum C, Martin P, Tamblyn R, et al. Reduction of inappropriate benzodiazepine prescriptions among older adults through direct patient education: the EMPOWER cluster randomized trial. JAMA internal medicine. 2014;174:890-8

53. Koegelenberg CF, Noor F, Bateman ED, et al. Efficacy of varenicline combined with nicotine replacement therapy vs varenicline alone for smoking cessation: a randomized clinical trial. JAMA. 2014;312:155-61

54. Ebbert JO, Hatsukami DK, Croghan IT, et al. Combination varenicline and bupropion SR for tobacco-dependence treatment in cigarette smokers: a randomized trial. JAMA. 2014;311:155-63

55. Taylor AH, Thompson TP, Greaves CJ, et al. A pilot randomised trial to assess the methods and procedures for evaluating the clinical effectiveness and cost-effectiveness of Exercise Assisted Reduction then Stop (EARS) among disadvantaged smokers. Health technology assessment 2014;18:1-324

56. Loebel A, Cucchiaro J, Silva R, et al. Lurasidone as adjunctive therapy with lithium or valproate for the treatment of bipolar I depression: a randomized, double-blind, placebo-controlled study. The American journal of psychiatry. 2014;171:169-77

57. Feder A, Parides MK, Murrough JW, et al. Efficacy of intravenous ketamine for treatment of chronic posttraumatic stress disorder: a randomized clinical trial. JAMA psychiatry. 2014;71:681-8

58. Wiles N, Thomas L, Abel A, et al. Clinical effectiveness and cost-effectiveness of cognitive behavioural therapy as an adjunct to pharmacotherapy for treatment-resistant depression in primary care: the CoBalT randomised controlled trial. Health technology assessment 2014;18:1-167

59. Herpertz-Dahlmann B, Schwarte R, Krei M, et al. Day-patient treatment after short inpatient care versus continued inpatient treatment in adolescents with anorexia nervosa (ANDI): a multicentre, randomised, open-label, non-inferiority trial. Lancet 2014;383:1222-9

60. Rose JE, Behm FM. Combination treatment with varenicline and bupropion in an adaptive smoking cessation paradigm. The American journal of psychiatry. 2014;171:1199-205
